# Newly Described Clinical and Immunopathological Feature of Dermatitis Herpetiformis

**DOI:** 10.1155/2012/967974

**Published:** 2012-06-03

**Authors:** Veronica Bonciolini, Diletta Bonciani, Alice Verdelli, Antonietta D'Errico, Emiliano Antiga, Paolo Fabbri, Marzia Caproni

**Affiliations:** ^1^Department of Critical Care, Section of Dermatology, University of Florence, Piazza Indipendenza, 11, 50129 Florence, Italy; ^2^Department of Clinical Physiopathology, University of Florence, 50139 Florence, Italy

## Abstract

Dermatitis herpetiformis (DH) is an inflammatory cutaneous disease with typical histopathological and immunopathological findings clinically characterized by intensely pruritic polymorphic lesions with a chronic-relapsing course. In addition to classic clinical manifestations of DH, atypical variants are more and more frequently reported and histological and immunological are added to them, whereas the impact on quality of life of patients with DH is increasingly important to a certain diagnosis. The aim of this paper is to describe all the possible clinical, histological, and immunological variants of DH in order to facilitate the diagnosis of a rare disease and, therefore, little known.

## 1. Introduction

Dermatitis herpetiformis (DH) is an inflammatory cutaneous disease with typical histopathological and immunopathological findings clinically characterized by intensely pruritic polymorphic lesions with a chronic-relapsing course, first described by Duhring in 1884 [1]. In 1966, Marks et al. [2]. reported small-bowel changes in patients with DH and later gastrointestinal abnormalities described in patients affected by DH were found to be the same as in those with celiac disease (CD) [3]. Currently, DH is considered as the cutaneous manifestation of gluten-dependent enteropathy, corresponding to CD.

DH is an autoimmune disease, a finding that is strongly supported by landmark studies revealing the granular deposition of immunoglobulin A (IgA) in the skin [4–6]. The same type of immunoglobulin was detected in the small intestinal mucosa of patient affected by CD, even before the development of the gluten-induced flat jejunal lesions. This observation on one hand emphasized the pivotal pathogenetic role of these immune deposits and from the other side represented a link between the two diseases [7, 8].

## 2. Clinical Features

The clinical morphology, in particular a polymorphous presentation and distribution of the lesions, are the hallmarks of the DH. Primary lesions of DH consist of grouped erythematous papules, urticarial plaques surmounted by vesicles or also blisters, which may be often replaced by erosions and excoriations, because of the intense itching characteristically associated with this condition. Chronic pruritus and excoriations might lead to lichenification, furthermore a transient postinflammatory hyperpigmentation may occur when the lesion resolve [9–13]. The symmetrical distribution of the herpetiform lesions on the extensor surfaces of the elbows (90%), knees (30%), shoulders, middle line of the back, buttocks, and sacral region is also a typical feature of the disease. In particular, Cottini [14]. In 1955 first described a clinical variant with exclusive localization of the lesions on the knees and elbows symmetrically. Anyway, scalp, nuchal area, face, and groin may be also involved. No clinical differences were described between darker- and white-skinned individuals, although DH remains primarily a prerogative of Caucasian population [15]. Most of the patients suffer not only itching but also tickle or burning sensation even before the onset of the skin lesions.

An uncommon skin manifestation of DH is represented by purpuric lesions occasionally described on palmo-plantar surfaces of children, but rarely reported in adults. First descriptions of DH presenting as purpuric, erythematous, or hemorrhagic palmar or plantar lesions date from 1971 [16, 17]. Fraser et al. reported such lesions in four of 14 patients with DH [17]. In 1979, Katz and Marks noted that vesicles may be hemorrhagic, particularly if they are located on the hands [18]. Moulin et al. [19] eventually described reported four DH patients with palmar “pseudopurpuric lesions” which showed typical histologic changes of DH in three out of four cases. Pierce et al. [20] described a 27-year-old man with typical DH and additional purpuric lesions on the palms. In 1986, 47 pediatric series of 47 DH cases by Karpati et al. [21], 30 (64%) showed red-brown palmar purpuric lesions, which, however, were not biopsied. Sometimes, petechial lesions on the fingertips may be the only symptom of DH, as reported by Moulin et al. [19], Rutten and Goos [22], Hofmann et al. [23], and recently Flann et al. [24]. Finally, the last case was described in 2011 by Heinlin et al. [25]. In a 15-year-old female with a 6-month history of recurrent painful petechiae on the fingers and feet, that was diagnosed as DH after histopathology, direct- and indirect- immunofluorescence (DIF, IIF).

However, atypical clinical presentation of DH reported in the literature includes also palmoplantar keratosis, wheals of chronic urticaria and lesions mimicking prurigo pigmentosa [26–28]. In particular, Ohshima et al. [26] in 2003 described a 63-year-old Japanese man which presented palmoplantar keratosis, in addition to itchy areas of erythema on the buttocks and knees with small blisters on the border that were clinically, histologically, and immunologically compatible with DH. The histologic evaluation of palmoplantar keratotic lesions showed hyperkeratosis, acanthosis, and cellular infiltration, mainly of lymphocytes, in the dermal papillae, aspects that were more compatible with psoriasis, but DIF showed granular IgA deposits in the dermal papillae confirming the diagnosis of DH. An unusual clinical presentation of DH in children described instead by Powell et al. [27] in 2004 consisted of chronic urticaria-like skin lesions. Histologic evaluation showed neutrophilic microabscesses in the dermal papilla with subepidermal blister formation, suggestive of DH, that was confirmed by fibrillar IgA deposition along the basement membrane zone (BMZ) with papillary accentuation revealed by DIF. Finally, Saito et al. [28] in 2005 described another atypical clinical presentation of DH, that was also in a Japanese subject, with features of prurigo pigmentosa but characterized by both histological and immunological aspect of DH.

## 3. Histopathology

The classic histopathologic features of DH seen on light microscopy include a subepidermal cleft with neutrophils, that are considered the most likely responsible for the dermal-epidermal separation [29], and a few eosinophils at the tips of dermal papillae, that are often accompanied by a perivascular mixed inflammatory infiltrate [12, 26]. While these findings are characteristic, there are a number of patients who present with pruritic, excoriated skin lesions with clinical and immunological features of DH in whom the histologic findings are nonspecific and do not confirm the diagnosis as showed by Warren and Cockerell [30], which found that 37.5% of DH patients had hematoxylin and eosin findings of a lymphocytic infiltrate only with fibrosis in the dermal papillae and ectatic capillaries. The authors hypothesized that the nonspecific histologic findings could represent both a sampling error on the part of the clinician taking the biopsy, in particular, choosing excoriated lesions that correspond to a later stage of the disease, or the pathology laboratory in sampling the lesion for histology, considering appropriate a progressive cutting of the tissue block, or distinct subgroup of dermatitis herpetiformis, possibly with a separate antigenic target [30].

## 4. DIF

Actually DIF of uninvolved skin collected in the perilesional site is considered as the diagnostic gold standard for DH [12]. The choice of normal appearing perilesional skin as site of biopsy specimen for DIF is not random, because in this site DH patients showed greater IgA deposition than in nonlesional or lesional skin as demonstrated by Zone et al. [4].

Two different patterns are possible: (a) granular deposits in the dermal papillae and (b) granular deposits along the basement membrane. In both cases, deposits are thought to be polyclonal but are mainly composed of IgA1 [31]. The two patterns may also be present as a combination resulting in granular IgA deposition along the basement membrane with accentuation at the tips of the dermal papillae [9–11, 32]. A third different pattern, presents in 50% of Japanese populations, first described in 1993 by Kawana and Segawa [33] as “fibrillar pattern,” was subsequently reconsidered by Ko et al. [34] in 2010. The fundamental difference between this last and previous is that the IgA deposition presents as linear streaks rather than fine granules in the papillary dermis. As suggested by Ko et al., the fibrillar pattern of IgA deposition may correlate with a clinical variant of DH or another as yet undefined disorder, and some case reports of atypical clinical presentations, that may be urticarial or psoriasiform, support this hypothesis [35, 36]. The correlation between the fibrillar DIF pattern and DH is, however, still debated. Although often the granular and the fibrillar patterns are associated in patients with DH, the last one alone more often correlates with atypical features, including atypical clinical presentations, absence of HLA-B8/DR3DQ2 haplotype, and lack of gluten-sensitive enteropathy or detectable circulating autoantibodies [37]. However, not all authors agree that, considering that the two different pattern may be the expression of a different method of sectioning [33].

## 5. Serologic Findings

Serologic testing is a useful adjunct to tissue-based studies. Contrary to the other bullous disorders, DH patients have no circulating autoantibodies binding to the cutaneous basement membrane components or to other adherent structures of the skin, but they have gluten-induced IgA autoantibodies against transglutaminase (TG) 2 and TG3 also called tissue-TG (t-TG) and epidermal-TG (e-TG), respectively [13].

TG represents an evolutionary conserved family of Ca^+2^-dependent enzymes that covalently cross-link or modify proteins by formation of an isopeptide bond between a peptide-bound glutamine residue and a primary amine, most commonly a lysine residue either within the same or a neighboring polypeptide chain [38]. Nine human types of TG were identified and some of them are expressed in the epidermis. TG2, among them, is the best characterized and also the most abundant and widely distributed [39]. Over time, different authors demonstrated that the enzymatic activity of TG2 is implicated in several diseases as Huntington disease, Alzheimer disease, and CD [40–42]. In particular, CD TG2 catalyzes highly specific deamidation of gluten peptides improving the binding of the peptides to the disease-associated HLA DQ2 and DQ8 molecules and becoming essential for the T-cell-mediated immune response against gluten. TG2 is also the primary autoantigen of CD recognized by autoantibodies of IgA1 class. These autoantibodies are considered the main serological marker of CD as disease-specific and more generally of gluten-sensitivity diseases and therefore of DH [12, 38, 43]. Levels of anti-TG2 correlate with bowel damage and gluten-free diet adherence in DH/CD patients and are measured using an enzyme-linked immunosorbent assay (ELISA) [44]. In DH, some authors have demonstrated an IgA anti-tTG specificity higher than 90%, and a sensitivity ranging from 47% to 95% [45–49]. However, as demonstrated by Sárdy et al. [50] the target autoantigen of DH is represented by TG3, that shares a 64% of homology with TG2 and overlapping sensitivity and specificity. As recently demonstrated by Stamnaes et al. [39] is also able to accommodate gluten peptides as substrates and form either thioester or isopeptide-linked complexes with these peptides stimulating the immune system's response, even if it is still unclear how it gets to the skin lesions. Although promising, the anti-eTG assay has not yet been approved in the United States for *in vitro* use to diagnose DH [51].

In addition to anti-TG2 antibodies, also those anti-EMA have become relatively sensitive and specific tools for initial detection of gluten-sensitive disease and, therefore, of DH. In particular, anti-EMA antibodies belong to the IgA1 subclass and are directed against primate smooth muscle reticular connective tissue. The detection of EMA is based on an indirect immunofluorescence assay on monkey oesophagus and it is more time-consuming and operator-dependent than the one of anti-TG2 ELISA testing [52], showing a specificity close to 100%, and a sensitivity ranging from 52% to 100% for the diagnosis of DH [44–47, 53].

Other autoantibodies are shared between DH and CD such as antigliadin antibodies and antireticulin antibodies, without, however, the same diagnostic value of anti-TG2 and anti-EMA.

In 2006, Sugai et al. first showed that the most reliable diagnostic test to identify gluten sensitivity in DH patients was the detection of antibodies against deamidated synthetic gliadin-derived peptides, both IgA and IgG isotypes [54]. Recently, a new CD serum marker based on TG2 covalently cross-linked to deamidated gliadin peptides, coined “neo-epitope,” was carefully studied by Matthias et al. and data supporting the use of this new assay (AESKULISA CeliCheck New Generation) in CD diagnosis were obtained in a longitudinal study of 2684 eligible subjects demonstrating a sensitivity of 92,31% and a specificity of 82,89%, that were highest when compared to TG2 ELISA test or anti-EMA tests [55]. The diagnostic value of these antibodies was also confirmed by Jaskowski et al. [56], which demonstrated the superior sensitivity of anti-tTG/DGP EIA screen (IgA + IgG) compared to IgA anti-tTG both in pediatric CD (92,6% versus 90,7%) and adult DH (65% versus 48,8% and 62% versus 44% in retrospective and prospective sera, resp.). Furthermore, very recently, Kasperkiewicz et al. [57] showed the higher sensitivity of the anti-GAF3X ELISA, a novel CD serologic assay using deamidated gliadin-analogous fusion peptides, to detect CD-associated autoantibodies in patients with DH compared with tests using native gliadin, tTG or endomysium as substrates.

## 6. Conclusion

From the data reported above, it seems evident that the diagnosis of DH can be difficult and, therefore, requires a complex approach, that should be clinical, histological and immunological, having regards to the atypical variants more and more frequently described in the literature. This becomes crucial since, as it is known, patients with DH present a significant reduction in their quality of life, mainly due to the need of a lifelong gluten-free diet and, consequently, to a significant change in lifestyle and eating habits.
